# Rapid screening for autoimmune diseases using Fourier transform infrared spectroscopy and deep learning algorithms

**DOI:** 10.3389/fimmu.2023.1328228

**Published:** 2023-12-15

**Authors:** Xue Wu, Wei Shuai, Chen Chen, Xiaomei Chen, Cainan Luo, Yi Chen, Yamei Shi, Zhengfang Li, Xiaoyi Lv, Cheng Chen, Xinyan Meng, Xin Lei, Lijun Wu

**Affiliations:** ^1^ Department of Rheumatology and Immunology, People’s Hospital of Xinjiang Uygur Autonomous Region, Urumqi, Xinjiang, China; ^2^ Graduate School of Xinjiang Medical University, Urumqi, Xinjiang, China; ^3^ College of Software, Xinjiang University, Urumqi, Xinjiang, China; ^4^ College of Information Science and Engineering, Xinjiang University, Urumqi, Xinjiang, China

**Keywords:** Fourier transform infrared spectroscopy, ankylosing spondylitis, rheumatoid arthritis, osteoarthritis, multiscale fusion, deep learning, diagnosis

## Abstract

**Introduce:**

Ankylosing spondylitis (AS), rheumatoid arthritis (RA), and osteoarthritis (OA) are three rheumatic immune diseases with many common characteristics. If left untreated, they can lead to joint destruction and functional limitation, and in severe cases, they can cause lifelong disability and even death. Studies have shown that early diagnosis and treatment are key to improving patient outcomes. Therefore, a rapid and accurate method for rapid diagnosis of diseases has been established, which is of great clinical significance for realizing early diagnosis of diseases and improving patient prognosis.

**Methods:**

This study was based on Fourier transform infrared spectroscopy (FTIR) combined with a deep learning model to achieve non-invasive, rapid, and accurate differentiation of AS, RA, OA, and healthy control group. In the experiment, 320 serum samples were collected, 80 in each group. AlexNet, ResNet, MSCNN, and MSResNet diagnostic models were established by using a machine learning algorithm.

**Result:**

The range of spectral wave number measured by four sets of Fourier transform infrared spectroscopy is 700-4000 cm-1. Serum spectral characteristic peaks were mainly at 1641 cm-1(amide I), 1542 cm-1(amide II), 3280 cm-1(amide A), 1420 cm-1(proline and tryptophan), 1245 cm-1(amide III), 1078 cm-1(carbohydrate region). And 2940 cm-1 (mainly fatty acids and cholesterol). At the same time, AlexNet, ResNet, MSCNN, and MSResNet diagnostic models are established by using machine learning algorithms. The multi-scale MSResNet classification model combined with residual blocks can use convolution modules of different scales to extract different scale features and use resblocks to solve the problem of network degradation, reduce the interference of spectral measurement noise, and enhance the generalization ability of the network model. By comparing the experimental results of the other three models AlexNet, ResNet, and MSCNN, it is found that the MSResNet model has the best diagnostic performance and the accuracy rate is 0.87.

**Conclusion:**

The results prove the feasibility of serum Fourier transform infrared spectroscopy combined with a deep learning algorithm to distinguish AS, RA, OA, and healthy control group, which can be used as an effective auxiliary diagnostic method for these rheumatic immune diseases.

## Graphical Abstract

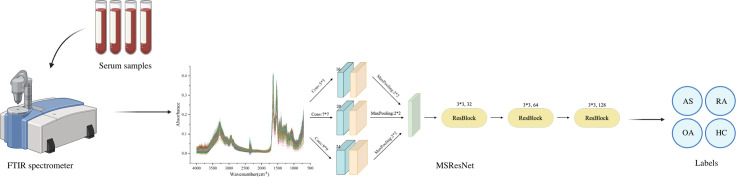

## 1 Introduction

Ankylosing spondylitis (AS), rheumatoid arthritis (RA), and osteoarthritis (OA) are three similar chronic inflammatory diseases. AS is an autoimmune disease that affects tendon attachment points, mainly manifesting as sacroiliac joint inflammation and chronic spondylitis (1). It is more prevalent in young men. Conversely, RA is a chronic autoimmune disease characterized by erosive arthritis (2), with a high prevalence in women. OA, meanwhile, is a degenerative joint disease with lesions in the cartilage (3), which presents with degenerative changes in the cartilage, secondary synovitis, bone metaplasia forming bony encumbrances, and, in more severe cases, causing cystic changes and destruction of the subchondral bone. It is a significant cause of disability in both the elderly and young people (4). These three rheumatic diseases are common musculoskeletal disorders worldwide, with the peak incidence of AS and RA occurring in young and middle age, where patients have arthritis, spinal stiffness, and deformity, ultimately leading to severe disability and, consequently, a substantial financial burden on families. According to the literature, osteoarthritis is the eleventh risk-ranked disability factor globally (5). The increasing prevalence of OA poses a substantial challenge to the health of middle-aged and elderly individuals. Since there is a lack of adequate clinical cures for AS, RA, and OA, early screening remains the only available option to alleviate the condition of patients.

In studies used to diagnose ankylosing spondylitis (AS), specific markers such as human leukocyte antigen B27 (HLA-B27) and C-reactive protein (CRP) are positive in 85-95% of AS patients (6). However, in most other autoimmune disease patients, they also were significantly positive (7, 8), indicating that they may not be reliable indicators for diagnosing AS or determining the effectiveness of treatment. The diagnosis of RA is based on the patient’s symptoms, test results, family history, and assessment of risk factors. For example, elevated CRP and ESR levels in serum tests (9) and the presence of RA-specific autoantibodies (10) can contribute to the diagnosis. Additionally, the use of ultrasound (11) and magnetic resonance imaging (MRI) (12) has been helpful in monitoring and diagnosing disease activity in RA patients. These diagnostic methods offer advantages such as relatively low cost, high usability, and real-time imaging capability (13). However, it depends on the operator’s skill and requires rigorous training in measurement and quality assessment. Currently, the gold standard for the diagnosis of OA mainly includes X-ray imaging (plain X-ray film), MRI, routine clinical examination of symptomatic joints (14), etc. X-ray imaging is considered safe, cost-effective, and widely accessible. However, radiographs are not very sensitive in detecting the early stages of OA (5), and interpreting the images requires a skilled practitioner.

Serological biomarkers and medical imaging are the primary diagnostic methods for three rheumatological immunological diseases: AS, RA, and OA. However, these methods have drawbacks, such as complexity, invasiveness, and reliance on the operator’s skills. It is, therefore, necessary to identify a simple, rapid, and non-invasive method to differentiate between AS, RA, and OA, as well as healthy controls, to diagnose these diseases early.

In recent years, the application of FTIR spectroscopy for the non-invasive, efficient, and rapid screening of rheumatic and immune diseases has gained attention from researchers (15, 16). Fourier transform infrared (FTIR) spectroscopy is a non-invasive, cost-efficient, and highly available technology (17). It assesses the individual biomolecules of a sample by analyzing the vibrational and rotational level changes in infrared absorption (18). FT-IR spectroscopy can measure differences in serum composition and detect abnormalities in specific molecules in proteins, lipids, nucleic acids, and other key markers of pathogenesis (19). FTIR was used for the diagnosis of many diseases and material testing. For instance, Francesco et al. (20) discovered microcalcifications in human ovarian plasma tumor tissues containing amorphous calcium carbonate phosphate, employing micro-Fourier transform infrared spectroscopy (micro-FTIR). Alla et al. (21) combined FTIR with colonoscopy and found from spectral analysis that spectral differences in the collagen fraction could be used for early and rapid screening of colorectal cancer. Studies have utilized FTIR to achieve earlier diagnosis of RA and OA, respectively (15, 16). Still, there is a lack of studies on the differentiation of similar rheumatic immune diseases. Highly accurate differentiation of AS, RA, OA, and healthy controls is vital for early treatment. However, owing to the low signal-to-noise ratio characteristic of most spectral signals (22), it is difficult to observe the differences between the spectra of several similar rheumatic diseases, likely leading to poor diagnosis. Therefore, designing a method to achieve high accuracy in diagnosing AS, RA, and OA disorders using FTIR is vital.

Machine learning is an approach to analyzing features through algorithms, learning the laws of data distribution, and making decisions or predictions based on specific tasks. With the continuous progress of computer technology research, the application of machine learning combined with FTIR spectroscopy in medical research is rapidly expanding. It can even replace the traditional methods used to diagnose various diseases. However, when the distinctions between the FTIR spectroscopy of the pieces are very small or even challenging to be observed by the human eye, the results achieved by simple machine learning algorithms cannot meet the diagnostic requirements, e.g., Support Vector Machines, Principal Component Analysis, K-Nearest Neighbors (23–25), and so on. Deep learning belongs to a class of methods in machine learning which solves many problems that traditional machine learning algorithms are ineffective at through its complex model structure. Deep learning methods combined with FTIR spectroscopy have been widely employed in disease screening. For example, Rose G et al. (26) utilized attenuated total reflection Fourier transform infrared (FTIR) spectroscopy to validate that the Wasserstein generative adversarial network enhancement method improves the accuracy of convolutional neural networks in distinguishing pancreatic cancer from non-cancerous samples. Hu et al. used convolutional neural networks combined with computed tomography (CT) to detect osteoporotic vertebral compression fractures (OVCF), achieving an accuracy of 81.70% on an independent test set (27). Wang et al. used a dual-mode model (MP-NN) to integrate serum metabolic fingerprints (SMFs) with protein tumor marker carcinoembryonic antigen (CEA) for the diagnosis of early lung adenocarcinoma and classification of lung nodules. Then based on MP-NN, the three-mode model MPI-RF, which uses random forest to integrate SMFs, CEA and image features, is superior to clinical diagnosis in the classification of pulmonary nodules (28). Yang et al. (29) classified the tissue transformation stages of esophageal squamous cell carcinoma with high accuracy based on a one-dimensional convolutional neural network (1-CNN) combined with micro-FTIR. Chen et al. (30) used an improved multi-scale fusion convolutional neural network on near-infrared spectral data to classify cumin and cumin with an accuracy of 100%. So far, most studies have been limited to classifying single diseases with healthy controls, and diagnostic models lack network improvement and comparison. For this reason, it is also interesting to design a deep learning model that is more suitable for classifying multiple rheumatologic diseases.

This study developed a multi-scale (MS) ResNet network structure based on FTIR spectra to accurately classify AS, RA, OA, and healthy controls. The model was designed to address two main challenges: substantial noise interference in the spectral measurement process and the high similarity among the three rheumatological diseases. To effectively handle these challenges, the model incorporates three multi-scale convolutional modules with different numbers of filters and convolutional kernel sizes. These modules are responsible for extracting features of different scales. Additionally, a residual block (ResBlock) is used to overcome the problem of network degradation. This block enables the extraction and fusion exploitation of local features, reducing noise interference in the spectral data and enhancing the model’s generalization ability. To ensure the superiority of the proposed MSResNet model, three other mainstream models (AlexNet, ResNet, and MSCNN) are selected for this experiment to compare the results.

## 2 Materials and methods

### 2.1 Sample preparation

This study obtained 80 serum samples each from AS, RA, OA, and healthy controls from the Department of Rheumatology and Immunology of Xinjiang People’s Hospital. The serum samples were centrifuged at high speed for 10 minutes at a temperature of 4 , and the supernatant layer was stored in a refrigerator at a temperature -80 overnight. During the assay, serum samples are thawed inherently at a consistent room temperature of 22 . Subsequently, 5 μL of the sample was pipetted onto the FTIR spectrometer. After 10 minutes of natural drying, the spectra were collected. The samples for this study were obtained from the People’s Hospital of Xinjiang Uygur Autonomous Region and ethical approval was obtained (KY2021101507).

### 2.2 Fourier transform infrared spectroscopy acquisition

The infrared spectra of serum were acquired by an FTIR spectrometer (FTIR-850, Gangdong Scientific, China), with air as the background, in the spectral range of 700-4000 cm^-1^, with 32 scans and a resolution of 4 cm^-1^. To reduce the noise interference in the acquisition process, all the samples were acquired once at three different positions, and the average value was taken as the infrared spectrum of that sample. Finally, 80 FTIR spectra of each of the serum samples from AS, RA, OA, and healthy control serum samples were obtained in this study, totaling 320 spectral samples.

### 2.3 Classification model

#### 2.3.1 AlexNet

**Figure 1:**
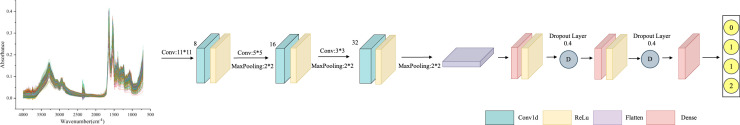
AlexNet framework structure diagram: consisting of three convolutional layers, with 32, 64, and 128 filters, and a kernel size of 3. After each layer, a batch normalization layer and a maximum pooling layer with a kernel size of 2 are connected, with a Dropout value of 0.4 to prevent model overfitting.

AlexNet is a feed-forward neural network established on Convolutional Neural Networks that mimics biology. It has convolutional computation and a deep structure (29) and is widely employed in image and natural language processing, among other areas. The network structure of AlexNet has convolutional primarily, pooling, and fully connected layers. The convolutional layer extracts information from the input data through filters (31), mapping local features from the previous layer to the next layer. The two parts of the convolutional layer, local connectivity, and weight sharing, dramatically reduce the number of parameters and the computational burden of complex nonlinear transformations. The AlexNet model framework proposed in this study is shown in **Figure 1**, which primarily includes three convolutional layers. The filters are 32, 64, and 128, respectively, and the kernel size is set to 3. Behind each layer, a batch normalization layer, and a max-pooling layer with a kernel of 2 are connected. The Flatten layer then one-dimensional the multi-dimensional input, reduce overfitting using the Dropout layer, and output the classification results through two Dense layers.

#### 2.3.2 ResNet

**Figure 2:**
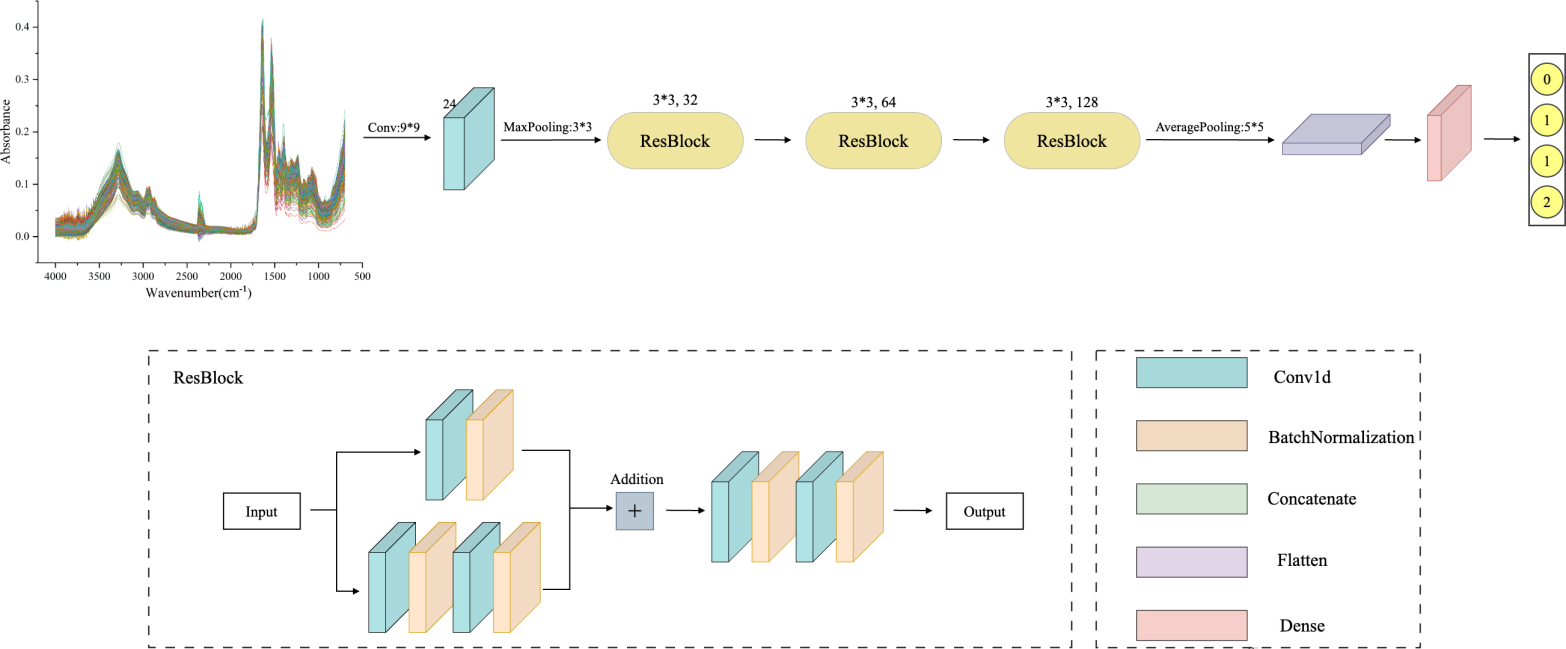
ResNet framework structure diagram: including one convolutional layer and three ResBlock layers, where the convolutional layer has a kernel size of 9, 24 filters, and a maximum pooling layer kernel size of 3. The convolution kernel size in all three ResBlock layers is 3, and the number of filters is 32, 64, and 128, respectively. Finally, using average pooling to extract overall features.

As deep learning models are applied in more complex domains, the depth of the network layers is increasing. During the backpropagation process, the gradient gradually diminishes, making the weight update of the shallow network almost ineffective. This increases training difficulty for the deep model, known as the gradient vanishing problem (32). To solve this, He et al. (33) proposed the ResNet model, which achieved significant results in the 2015 ImageNet image recognition competition. The ResNet model is based on the traditional CNN model and introduces the “residual block”. This block allows information to bypass one or more layers in the network, reaching the output directly. The ResNet model enables faster convergence and better performance by allowing the network to learn the residual function and make minor parameter updates. The structure of the Residual Block (ResBlock) used in this experiment is shown in **Figure 2**. The main branch consists of two convolutional layers and a Batch Normalization layer. There is also a shortcut branch, which has only one convolutional layer, and its parameters are the same in each ResBlock structure. Finally, the extracted multi-scale features are fed into the Flatten layer and classified by the Dense layer.

#### 2.3.3 MSCNN

**Figure 3:**
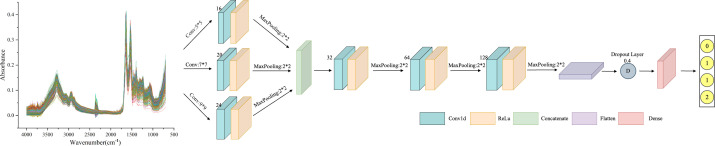
MSCNN framework structure diagram: including three parallel receptive fields, using convolution kernels of sizes 3, 7, and 9 to extract features of different scales. Each parallel convolution layer consists of Conv1d layers, BatchNormalization layers, and pooling layers. Integrate multi-scale features in the Concatenate layer.

As a particular type of neural network that incorporates convolutional computations, CNN has a strong capability for feature extraction. The network’s structure, characterized by parameter sharing and local connectivity, helps to reduce computational load, thereby enhancing CNN’s generalization ability in various application domains. However, traditional CNN models tend to lose important information from the original data during training (34). Additionally, extracting multi-scale features proves challenging, resulting in reduced efficiency. MSCNN builds on the concept of multicolumn DNN to extract multi-scale signals. This is achieved using three parallel convolutional layers with varying sensory field sizes. The structure of these three convolutional layers remains the same, comprising a Conv1d layer, a BatchNormalization layer, and a pooling layer. The multi-scale features are then merged in the Concatenate layer. Furthermore, the features extracted by the three convolutional layers are inputted to the Flatten layer, with overfitting addressed by incorporating the Dropout layer. Finally, the classification results for the spectral data are obtained through the two Dense layers. The MSCNN network framework is shown in **Figure 3**.

#### 2.3.4 MSResNet

**Figure 4:**
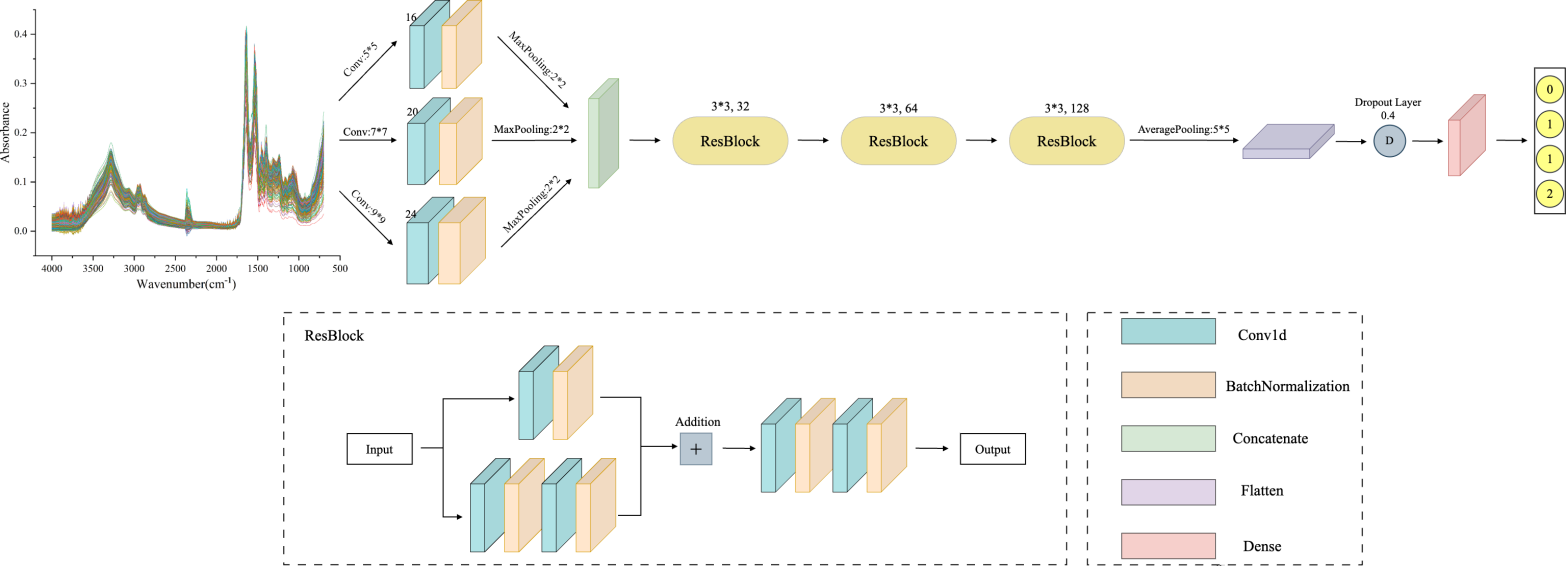
MSResNet framework structure diagram: using three convolutional kernels of different sizes instead of a single kernel to extract feature information at different scales. Then input the fused multi-scale features into the ResBlock block to improve model performance.

The MSResNet model proposed in this paper is designed based on ResNet, concerning the MSCNN model feature extraction, combining multi-scale information extraction with a residual structure to achieve a high-accuracy diagnosis of diseases. This multi-scale convolutional kernel design has been proven to work better in fields such as image recognition (35, 36). The MSResNet network framework is shown in **Figure 4**, where three convolutional kernels of different sizes are used instead of a single convolutional kernel to extract feature information at different scales. The fused multi-scale features are input into the ResNet structure to enhance the model implementation using the unique residual design.

### 2.4 Spectral analysis

**Figure 5:**
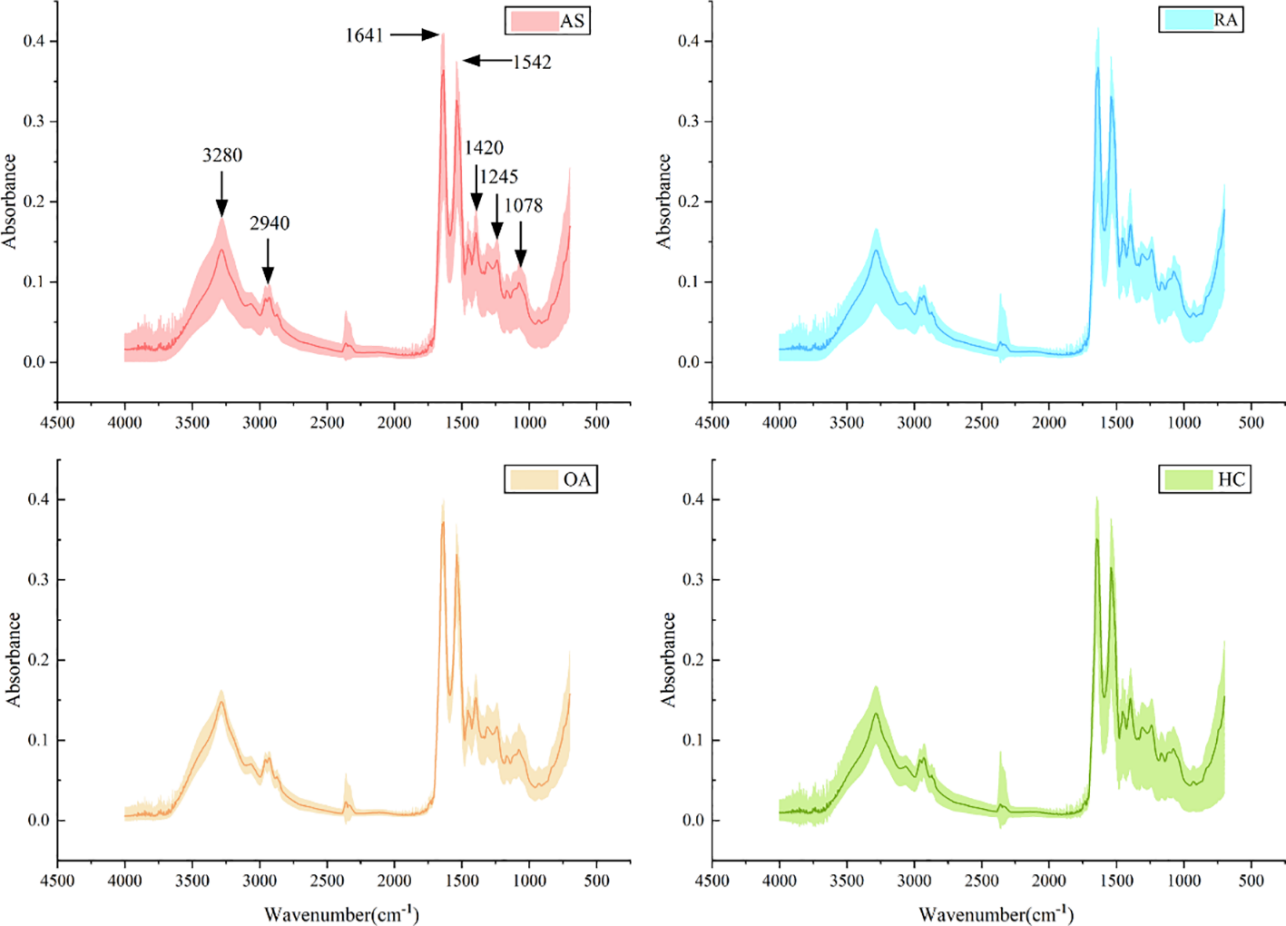
Average spectra (lines) and area of spectral regions (shaded) for all samples for ankylosing spondylitis (AS), rheumatoid arthritis (RA), osteoarthritis (OA), and healthy control (HC) serum samples.

**Figure 6:**
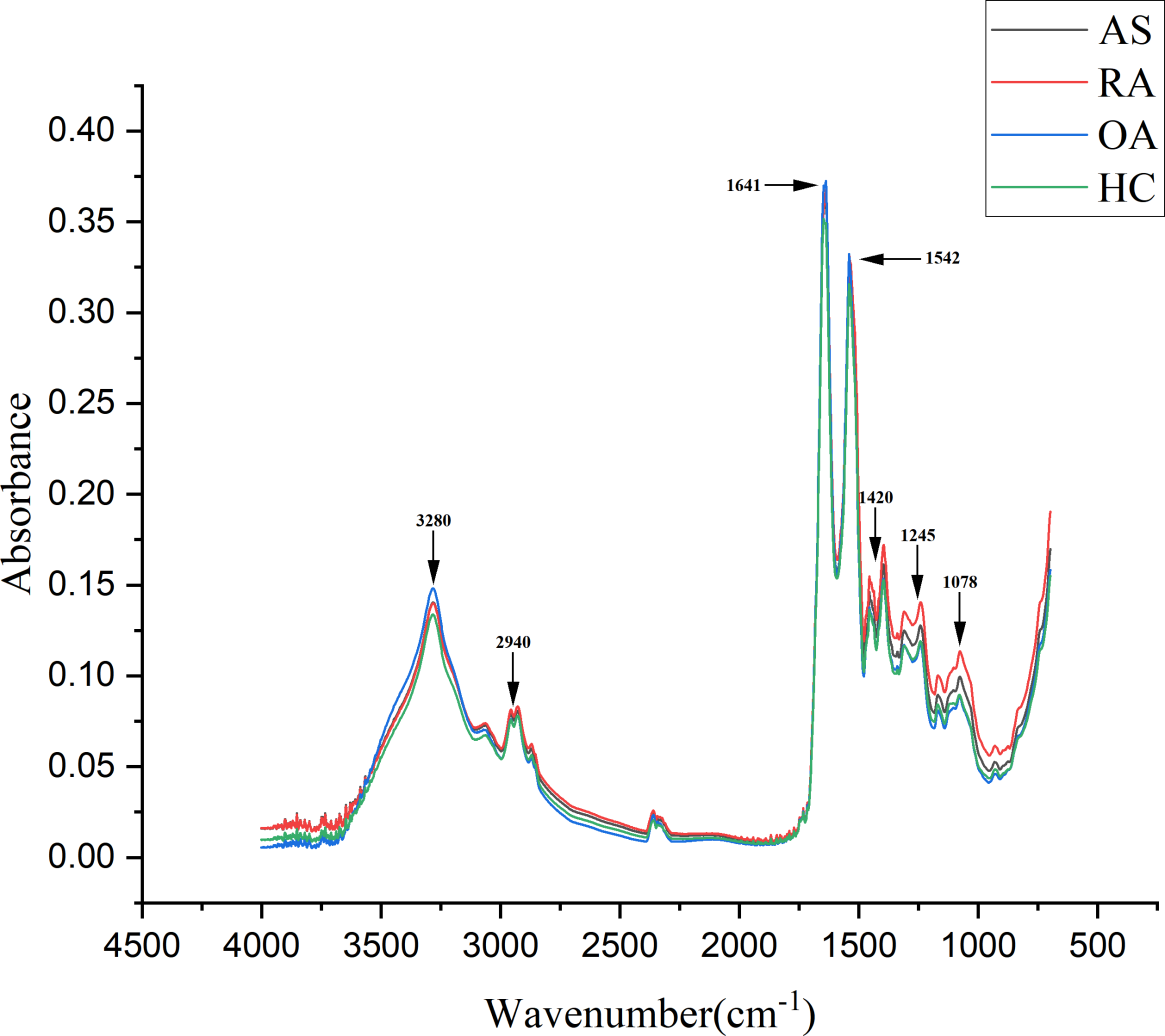
Comparison of average spectra of serum samples from ankylosing spondylitis (AS), rheumatoid arthritis (RA), osteoarthritis (OA), and healthy controls (HC).

A total of 320 patients were included in this study, including 80 patients in the AS, RA, OA, and HC groups, with an average age of 40.5 ± 6.0 years old and a male to female ratio of 1.1:1. There was no statistically significant difference in age and gender among the four groups, as shown in **Table 1**. **Figure 5** shows FTIR spectra of serum samples from AS, RA, OA, and healthy controls. The measured spectral wave number interval is 700-4000 cm^-1^, a wave number range containing a wealth of information on biomolecular fingerprints, such as proteins, lipids, carbohydrates, and nucleic acids in serum, identifying the disease category. The spectral curves of the four types are similar, with the primary distinction being the magnitude of the curve fluctuations. **Figure 6** shows the average spectral comparison of serum samples from three types of diseases and healthy controls (HC). The characteristic peaks of serum spectra are mainly at 1078, 1245, 1400, 1542, 1641, 2940, and 3280 cm^-1^, and **Table 2** lists the tentative material assignments for these major FTIR peaks. The most substantial characteristic peaks are at 1641 cm^-1^ (amide I) and 1542 cm^-1^ (amide II) (37, 38), which represent the stretching and bending vibrations on the amide C=O as well as the N-H groups of proteins, respectively (39), the broadband at 3280 cm^-1^ also corresponds to the N-H group, but it is a stretching vibration (40) and is referred to as the amide A mode; the vicinity of 1420 cm^-1^ represents the region of proteins, phosphate molecules, and fatty acids (16), and it has been illustrated that this wavelength is related to proline and tryptophan in proteins; 1245 cm^-1^ represents amide III, the asymmetric P=O stretching in PO_2_ (25); 1078 cm^-1^ represents the region of carbohydrates (15); and the area of 2940 cm^-1^ is dominated by fatty acids and cholesterol, among other substances (41).

**Table 1:** Age, gender, and statistical information of the samples.

Indicator	AS group (80)	RA group (80)	OA group(80)	HC group	χ^2^/t	P
Age (years)	39.4 ± 8.2	39.8 ± 5.9	41.6 ± 3.2	40.8 ± 5.2	2.149	0.094
Gender (female/male)	32/48	38/42	43/37	42/38	3.741	0.291

**Table 2:** Peak positions and tentative assignments of major FTIR bands.

Wavenumber (cm^-1^)	Corresponding substance
3280	the N–H group, a stretching vibration called the amide A mode
2940	Fatty acids and cholesterol
1641	Amide I, protein and stretching and bending vibrations on the amide C=O
1542	Amide II, stretching and bending vibrations on the N–H groups
1420	proline and tryptophan
1245	Amide III, asymmetric P=O stretching in PO_2_
1078	Carbohydrate region

### 2.5 Experimental setup

This experimental dataset consists of Fourier transform infrared spectra of serum samples from patients with AS, RA, OA, and healthy controls, and the four types of data were classified by AlexNet, ResNet network, multi-scale convolutional neural network, and multi-scale ResNet network. In addition, most of the typical medical studies of infrared spectroscopy are based on small datasets, insufficient training data can result in providing less valid information, and model training is prone to overfitting. To alleviate overfitting, five-fold cross-validation is used in the training process to improve the model’s generalization ability. The data set is divided into the training set and the test set in a 7:3 ratio, and the training set is used for cross-validation. It is randomly divided into five, four for training and one for validation. The training process batch size was set to 8, epoch to 200, optimizer to Adam, and learning rate to 0.0001. To compare the performance of the different models more intuitively, the experiments were compared by the subjects’ work characteristic curve (ROC) and area under the curve (AUC), accuracy, sensitivity, precision, and specificity metrics. ROC is a probability curve that shows the classification ability of a model curve. The AUC value is expressed as the area under the ROC curve; a larger AUC indicates a better classification model performance.

### 2.6 Classification results

**Figure 7:**
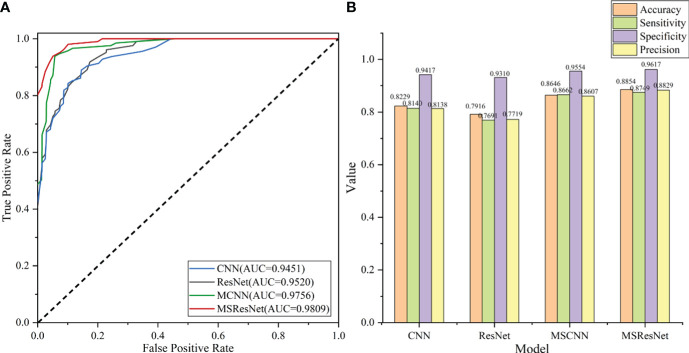
**(A)** plots the ROC curve of the model, and the AUC value represents the area under the ROC curve. The larger the AUC value, the better the generalization performance of the model. **(B)** shows the four evaluation index values of accuracy, sensitivity, precision, and specificity of model classification. The higher the accuracy value, the better the classification performance of the model.

The classification results of the four models, namely AlexNet, ResNet, MSCNN, and MSResNet, are presented in **Figures 7A, B**. **Figure 7A** displays the ROC curves of the models, with all models achieving AUC values exceeding 0.9. The highest AUC value, 0.98, is obtained by MSResNet. **Figure 7B** illustrates the accuracy, sensitivity, precision, and specificity metrics of the models’ classification. The specificity values for all four models exceed 0.9. However, the other three metrics, namely accuracy, sensitivity, and precision, show poorer results for AlexNet and ResNet compared to MSResNet. MSResNet demonstrates the best performance across all metrics, with values of 0.8854, 0.8749, 0.8829, and 0.9617 for accuracy, sensitivity, precision, and specificity, respectively. To further evaluate the models’ classification on the four types of samples, **Table 3** presents the classification accuracy and average value for each model. MSResNet achieves the highest average accuracy of 0.8749 and performs better in classifying the two types of samples, AS and HC. Comparing the evaluation metrics of all the models, it can be concluded that MSResNet exhibits the most effective classification for the four types of samples, namely AS, RA, OA patients, and Healthy Controls.

**Table 3:** Accuracy and mean values of the classification of the model for the four types of AS, RA, OA patients, and HC, with best results in bold.

Model	AS	RA	OA	HC	Average accuracy
AlexNet	0.7037	0.7059	**0.9615**	0.8846	0.8139
ResNet	0.7778	0.5294	**0.9615**	0.8077	0.7691
MSCNN	0.8519	**0.8824**	0.8462	0.8846	0.8662
**MSResNet**	**0.8889**	0.7647	0.8846	**0.9615**	**0.8749**

## 3 Discussion

AS, RA and OA are all common chronic inflammatory diseases that cause joint and bone abnormalities, often resulting in severe disability. They are the leading causes of chronic disability worldwide. However, if diagnosed early and treated promptly, the likelihood of disability or life-threatening damage can be greatly reduced. Unfortunately, there is currently no validated and unambiguous method to differentiate between AS, RA, OA, and healthy individuals. Therefore, there is a critical need for affordable and reliable detection methods, particularly in the early stages of these diseases. In vibrational spectroscopy, the chemical bonding of molecules in a sample can be examined without the need for labeling (42). This technique enables the distinction between diseased and healthy individuals by identifying specific wavefronts that are believed to be associated with disease specificity. Through the analysis of these distinct peaks, it is possible to identify disease-related chemicals, including proteins, nucleic acids, carbohydrates, and lipids. These specific regions in the spectrum can be considered as unique “fingerprints” of the disease. Overall, vibrational spectroscopy holds promise as a potential method for the early detection of AS, RA, and OA, as it allows for non-invasive and accurate identification of disease-specific markers (43). In this study, the first attempt was made to design an MSResNet network structure based on a multiscale and residual structure to efficiently differentiate sera from AS, RA, and OA patients as well as healthy controls by Fourier transform infrared spectroscopy and spectral analysis. By comparing with three other mainstream models (AlexNet, ResNet, MSCNN), MSResNet was found to have better diagnostic performance.

During the experiment, common and specific discriminatory waveforms were identified for the four samples through spectral analysis (**Table 1**). The common waveforms were observed because these diseases share certain common features and are all chronic joint inflammatory diseases. The specific waveforms were not readily apparent in the analysis, and the main difference among the four samples was the peak size of the common waveforms. For example, peaks were observed at 1641 cm^-1^ (amide I), 1542 cm^-1^ (amide II), 3300 cm^-1^ (amide A), 1420 cm^-1^ (proline and tryptophan), 1245 cm^-1^ (amide III), 1078 cm^-1^ (carbohydrates), and 2940 cm^-1^ (fatty acids and cholesterol). These substances have been identified in previous studies as signaling molecules for cell growth, differentiation, and apoptosis. It has been demonstrated that they induce synovial cell apoptosis by modulating diverse signaling pathways (44). Additionally, using electrospray ionization mass spectrometry for synovial tissue analysis, it has been found that amide levels are elevated in patients with RA and OA, and reduced in patients with AS (45). The relative intensity increase observed between the Raman bands located at 1241 cm^-1^and 1269 cm^-1^ (amide III doublet) by Takahashi et al. may be associated with structural changes under type II collagen loading. This suggests a higher content of disordered collagen in the cartilage of osteoarthritis (OA) patients (46). This is indeed evidence of collagen defects leading to abnormal cartilage structure. This finding indicates that current spectroscopic methods may contribute to identifying and quantitatively assessing the early manifestations of osteoarthritis. In addition, a study on non-targeted lipidomics analysis of synovial fluid and serum from rheumatoid arthritis (RA) patients at different disease activities and clinical stages (from pre-clinical to active to sustained remission) revealed that the lipidomic profile in RA joint fluid is correlated with the degree of inflammation and the severity of synovitis. Changes in amide levels can predict the therapeutic response to drugs (47). These results suggest that monitoring amide levels may aid in disease identification, predicting the evolution from pre-clinical to definitive disease, and assessing disease activity and treatment outcomes.

This study also revealed significant differences in four groups at 1420 cm^-1^ (proline and tryptophan). Amino acid metabolism is considered a key regulator of both innate and adaptive immune responses (48). Proline, abundant in the body and second only to glutamine and alanine, constitutes 50% of collagen in the body, approximately 30% of the body’s total protein. As collagen is a major component of cartilage, it plays a crucial role in constructing the cartilage tissue framework, supporting joint loading, and protecting and repairing damaged joint cartilage. Abnormal proline metabolism can lead to a reduction in collagen, resulting in decreased resistance of connective tissues, abnormal bone collagen metabolism, and the onset of chronic orthopedic diseases such as OA and RA. Hydroxyproline, a metabolic product of proline, serves as a marker for collagen degradation (49). On the other hand, tryptophan, an essential amino acid in the human body (50), has metabolites with immune, metabolic, and neuroregulatory functions in biology, making it a therapeutic target for various diseases. As early as the late 1950s, scholars proposed the use of the tryptophan content in synovial fluid to distinguish between inflammatory and non-inflammatory joint diseases. This is particularly relevant in the tryptophan metabolism pathway mediated by rate-limiting enzymes indoleamine-2,3-dioxygenase1(IDO1), indoleamine-2,3-dioxygenase2(IDO2), tryptophan-2,3-dioxygenase (TDO), and kynurenine monooxygenase (KMO). Studies have found that changes in serum tryptophan are closely related to disease progression, with the progression of RA leading to decreased tryptophan levels due to IDO1-induced tryptophan degradation (51), providing important theoretical basis for identifying diagnostic and therapeutic biomarkers for RA. In OA patients, increased protein hydrolysis may produce more free tryptophan in the intestines (52), leading to an overall increase in tryptophan and its metabolites. Additionally, research indicates that tryptophan metabolites indole-3-acetaldehyde and indole-3-acetic acid are involved in the occurrence and development of spondyloarthritis (SPA) (53). These research findings suggest that abnormal proline and tryptophan metabolism play crucial roles in the pathological mechanisms of RA.

Finally, this study observed differences in the peak at 2940 cm^-1^ (fatty acids and cholesterol) among the four groups, consistent with previous research. Studies have demonstrated the involvement of lipid metabolism in the onset of RA and AS. Abnormalities in lipid metabolism in immune cells contribute to the invasion and migration of synovial tissues in RA patients, promoting synovial inflammation, as well as cartilage and bone erosion. In RA patients, the synovium shows significantly higher levels of palmitic acid, total saturated fatty acids, long-chain MUFA, and/or total MUFA compared to osteoarthritis and healthy control groups. Conversely, in AS patients, the concentrations of many lipids decrease (54, 55). In summary, the above studies indicate that FTIR optimized with machine learning algorithms could serve as a convenient, rapid, and economical detection method for monitoring changes in substances such as amino acids and lipids in patients. This approach holds promise for disease diagnosis and prognosis research.

Many studies have reported the application of spectroscopic techniques in rheumatic diseases. Lee et al. (56) measured by Raman micro spectroscopy that the mineralization of subchondral trabecular bone (SCTB) tissues in osteoarthritis regions in knee osteoarthritis was markedly lower than that of the corresponding regions in control individuals, further demonstrating the potential value of SCTB for targeted therapies in OA. Cao et al. (57) utilized a multivariate dimensionality reduction method and a machine learning algorithm to analyze the correlation between spectral differences and clinical and immunological manifestations in RA patients. Prada et al. (41) developed the first prediction model for LDA based on FTIR with diagnostic accuracies of 97% and 85% for two diseases, namely Crohn’s disease (CD) and spondylarthritis (SpA), respectively. Collection of blood samples is non-invasive and simple, therefore spectroscopy-based disease diagnosis is easily reproducible and cost-effective. To this end, this experiment designed a multiscale combined residual block MSResNet model based on FTIR spectra to achieve high-performance classification of AS, RA, OA, and HC. The model effectively combines the multiscale module in a multiscale convolutional neural network with the ResNet model. The multiscale module consists of three convolutional layers with different numbers of filters and convolutional kernel sizes, which facilitates the extraction of multiscale and multilevel features. On the other hand, the residual block in the ResNet model avoids the deep model network degradation problem. With these designs, the MSResNet model achieves the extraction and fusion of local features of the four samples, which mitigates the effects brought by the noise of spectral data to enhance the generalization ability of the model diagnosis.

## 4 Conclusions

In this paper, we propose a robust method capable of differentiating between patients with AS, RA, and OA, as well as healthy controls. Firstly, the FTIR of the four serum samples was measured using a spectrometer, and then a multiscale residual convolutional neural network (MSResNet) was constructed to classify the spectral data. The model mainly consists of a multiscale module and a residual block, the multiscale module uses three sets of convolutional layers with different specifications to extract richer multiscale feature information, followed by a residual block used to solve the network degradation problem brought by the simple deep model. In addition to this, this study also conducts comparison experiments with three other models (AlexNet, ResNet, and MSCNN). The experimental results show that using a multi-scale feature fusion model outperforms the traditional ResNet framework that extracts features using only one scale, and the multi-scale combined residual block model also outperforms the simple MSCNN model. This fully demonstrates the superiority of our model for high similarity spectral classification, which enables non-invasive, fast, and low-cost identification of four types of data, namely ankylosing spondylitis, rheumatoid arthritis, osteoarthritis, and healthy control group, by extracting their multi-scale and multi-level features from the spectral data. The spectral analysis also revealed that amides, proline, and tryptophan are likely to be spectral “biometric fingerprints”, and thus FTIR may be a promising tool for the study of rheumatic diseases as a rapid, low-cost, and accurate biomarker identification method, and may also provide effective information for prognostic examinations. Although this article has detailed and validated the effectiveness of using FTIR method to diagnose several autoimmune diseases, early detection systems using FTIR combined with deep learning algorithms still require deeper and more extensive validation for treating other diseases.

## Data availability statement

The data analyzed in this study is subject to the following licenses/restrictions: Due to the nature of this research, participants of this study did not agree for their data to be shared publicly, so supporting data is not available. Requests to access these datasets should be directed to Xue Wu,wuxue199094@163.com.

## Ethics statement

The studies involving humans were approved by Ethics Committee of Xinjiang Uygur Autonomous Region People’s Hospital. The studies were conducted in accordance with the local legislation and institutional requirements. The participants provided their written informed consent to participate in this study.

## Author contributions

XW: Data curation, Investigation, Methodology, Writing – original draft, Writing – review & editing. WS: Methodology, Software, Writing – original draft. CC: Data curation, Supervision, Writing – review & editing. XC: Data curation, Formal Analysis, Writing – review & editing. CL: Writing – review & editing. YC: Formal Analysis, Supervision, Writing – review & editing. YS: Project administration, Resources, Writing – review & editing. ZL: Formal Analysis, Investigation, Writing – review & editing. XYL: Resources, Supervision, Writing – review & editing. CgC: Methodology, Resources, Supervision, Writing – review & editing. XM: Formal Analysis, Validation, Writing – review & editing. XL: Writing – review & editing. LW: Methodology, Resources, Supervision, Writing – review & editing.
